# Is hemorrhage in acute reperfused myocardial infarction a new marker for the severity of tissue injury?

**DOI:** 10.1186/1532-429X-11-S1-P65

**Published:** 2009-01-28

**Authors:** Andreas Kumar, Jordin D Green, Jane M Sykes, Andrea Mitchell, Gerald Wisenberg, Matthias G Friedrich

**Affiliations:** 1 University of Calgary, Calgary, AB Canada; 2 University of Western Ontario, London, ON Canada

**Keywords:** Cardiovascular Magnetic Resonance, Reperfusion Injury, Microvascular Obstruction, Late Enhancement, Cardiovascular Magnetic Resonance Study

## Introduction

Reperfusion injury in myocardial infarction leads to microvascular obstruction, which can occur with or without gross reperfusion hemorrhage. The incidence and implications of reperfusion hemorrhage are not well investigated. A recently described *in vivo* imaging approach using T2*-weighted cardiovascular magnetic resonance can help investigate the pathophysiology of reperfusion hemorrhage *in vivo*.

## Hypothesis

We hypothesized that hemorrhage reflects a severer from of reperfusion injury and therefore occurs with larger infarct size and worse LV function as compared to reperfusion injury without hemorrhage.

## Methods

In 14 female mongrel dogs, myocardial infarction was induced by ligation of the left anterior descending coronary artery for 2–4 hours, followed by reperfusion. On day 3 ± 1, a cardiovascular magnetic resonance study was performed in vivo to (1) assess presence of microvascular obstruction, defining reperfusion injury (2) assess presence of reperfusion hemorrhage (3) quantify left ventricular end-diastolic volume, ejection fraction and cardiac output and (4) quantify infarct size with late enhancement. An independent-samples t-test was performed to compare these parameters in dogs with and without hemorrhage in reperfusion injury.

## Results

From 14 dogs, 9 had microvascular obstruction, and 4/9 had reperfusion hemorrhage in addition to microvascular obstruction.

Dogs with hemorrhagic infarcts had significantly larger infarct size (26.1 ± 6.6 g vs. 5.5 ± 3.9 g, p < 0.05), lower LV ejection fraction (28 ± 7% vs. 53 ± 12%, p < 0.05), and lower cardiac output (1.9 ± 0.2 l/min vs. 2.8 ± 0.5 l/min, p < 0.05). There were no differences in LV end-diastolic volume and LV mass.

## Discussion and conclusion

In this dog model of ischemia/reperfusion injury, gross hemorrhage was associated with significantly larger infarct size and worse LV functional parameters. This supports the hypothesis that hemorrhage may occur with advanced, severe ischemic tissue injury only.

